# Scleritis in Takayasu Arteritis

**DOI:** 10.7759/cureus.37724

**Published:** 2023-04-17

**Authors:** Swetha Chittipolu, Jennifer L Kennard, Rajachendra S Tumma, Amy R Doyle

**Affiliations:** 1 Internal Medicine, North Mississippi Health Services, Tupelo, USA; 2 Rheumatology, North Mississippi Health Services, Tupelo, USA

**Keywords:** immunosuppression, narrowing of aorta, large vessel vasculitis, takayasu arteritis, scleritis

## Abstract

Takayasu arteritis (TA) is a chronic vasculitis of unknown etiology which primarily affects the aorta. The manifestations of this disease include secondary hypertension, reduced pulses, limb claudication, discrepant blood pressure, arterial bruits, and heart failure due to aortic insufficiency or coronary artery disease. The ophthalmological findings are late manifestations. Here, we present a case of a 54-year-old woman who presented with scleritis of the left eye. She sought care with an ophthalmologist and was treated with topical steroids and non-steroidal anti-inflammatory drugs (NSAIDs) with no relief. She then received oral prednisone with symptom amelioration.

## Introduction

Takayasu arteritis (TA) is a chronic giant cell vasculitis that predominately affects young women. Up to 25% of cases occur before the age of 20 years. The disease involves panarteritis with inflammatory mononuclear cells and occasionally giant cells. The vasa vasorum is frequently involved [1]. Carotid artery tenderness, limb claudication, presyncope, headache, and weakening of pulses are the most frequent clinical features; less common presentations can include ocular manifestations [2]. Diagnosis is made from clinical presentation, relevant laboratory values, and imaging. A biopsy is not necessary to diagnose TA. Management includes glucocorticoid therapy, adjunctive immunosuppressants, and rarely surgical intervention. Scleritis is a rarer presentation but a known feature of TA [3].

## Case presentation

**Figure 1:**
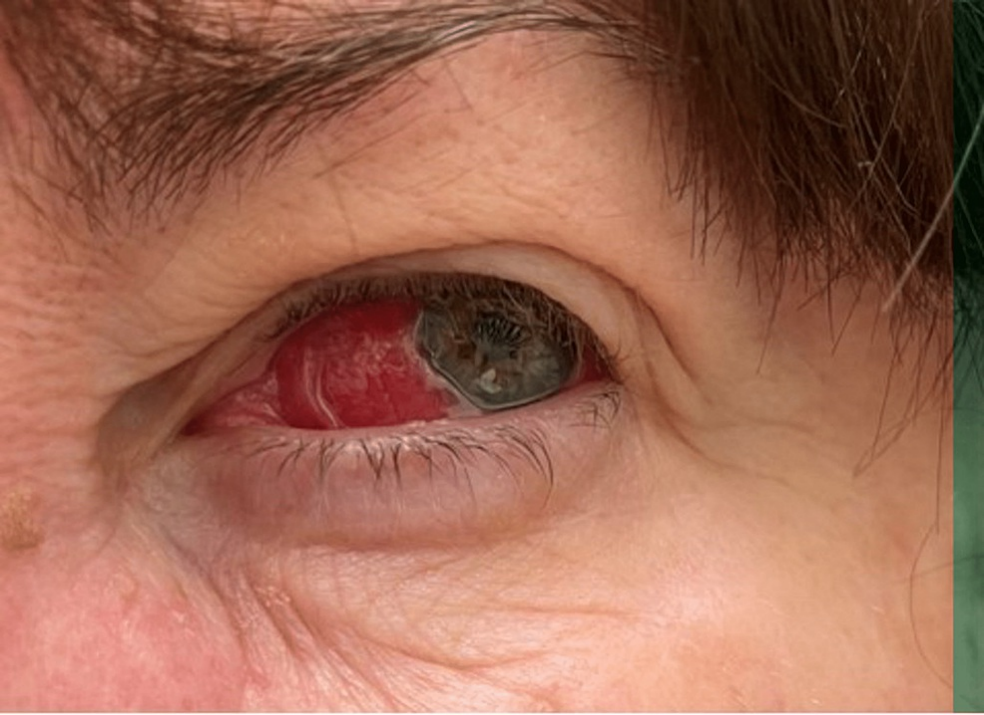
Left eye scleritis prior to oral prednisone

A 54-year-old Caucasian woman with hypertension presented with left eye pain and injection (Figure 1), and right arm limb claudication. She was evaluated by an ophthalmologist who diagnosed her with scleritis. Topical steroids and systemic non-steroidal anti-inflammatory drugs (NSAIDs) provided little relief. Later, she was seen in the rheumatology clinic. She reported discrepant blood pressure in her arms that started in her late 20s and was found to have narrowed blood vessels on the right. She did not seek further care due to the lack of insurance. A review of systems was notable for chronic weakness and heaviness in the right arm provoked by activity, and fatigue.

Physical examination revealed left arm blood pressure of 210/76, right arm blood pressure of 150/74, right carotid bruit +, right arm diameter smaller than the left arm, and weak pulse in her right arm. Investigations revealed an elevated C-reactive protein (CRP) of 1.3 and an elevated erythrocyte sedimentation rate (ESR) of 23. Complete blood count and comprehensive metabolic panel were within normal limits (Table 1).

**Table 1:** Laboratory analysis CRP: C-reactive protein; ESR: Erythrocyte sedimentation rate; CBC: Complete blood count; CMP: Comprehensive metabolic panel; WNL: Within normal limits

Test	Results
CRP	1.3
ESR	23
CBC & CMP	WNL

**Figure 2:**
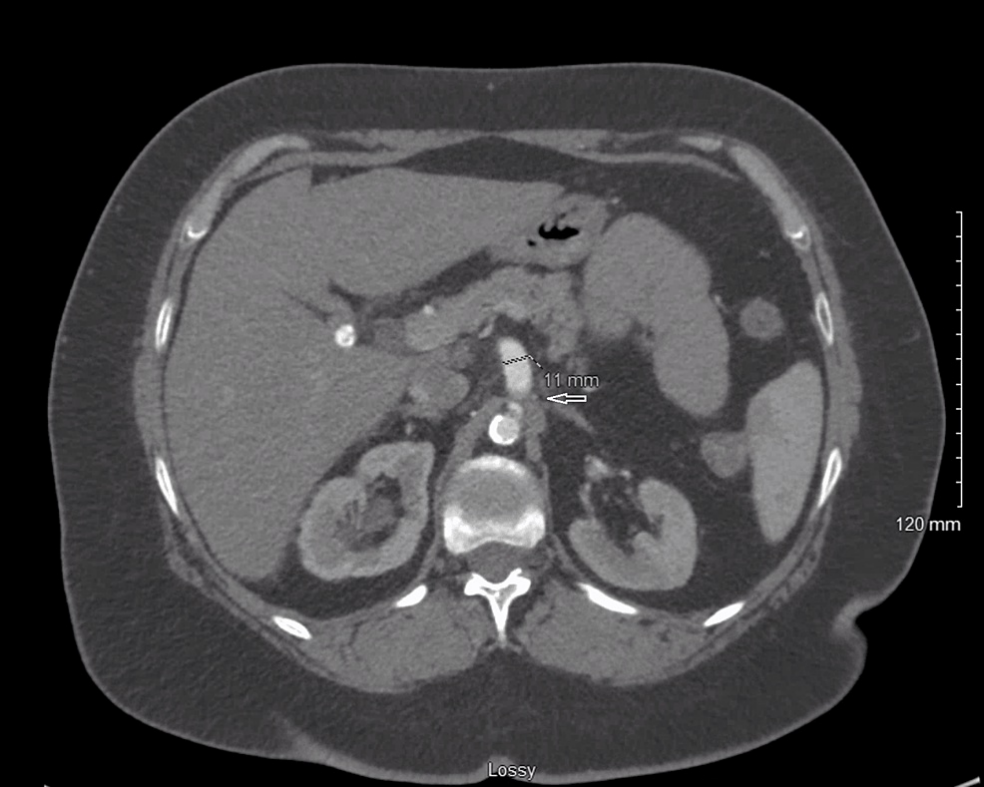
CT abdomen showing narrowed celiac artery

Imaging findings include transthoracic echocardiography showed a normal left ventricular size with an estimated ejection fraction of 55%, trace mitral regurgitation, and a structurally and functionally normal aortic valve. Computed tomography angiography (CTA) chest and abdomen showed narrowing of the aorta, celiac artery ostium, bilateral renal artery, and stenosis of R axillary with the brachiocephalic artery (Figure 2).

**Figure 3:**
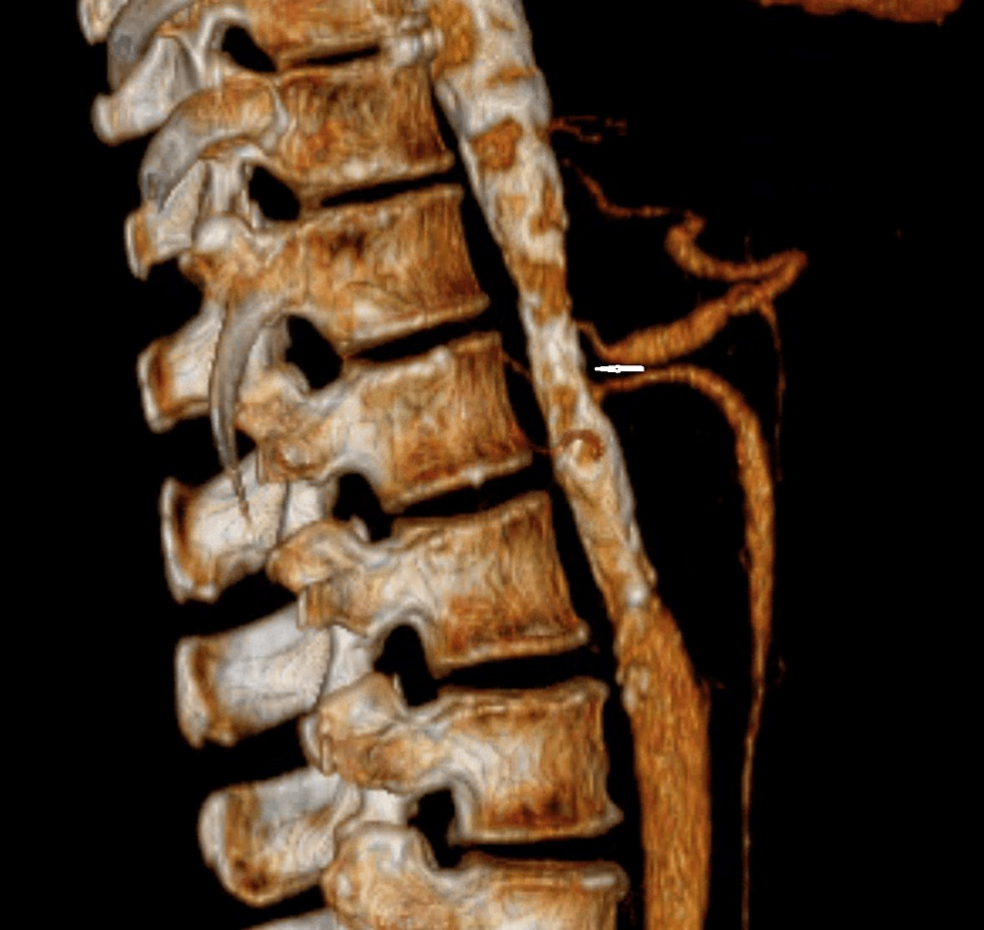
MRA abdomen showing narrowed abdominal aorta MRA: Magnetic resonance angiography

 Magnetic resonance angiography (MRA) abdomen showed narrowed abdominal aorta and celiac artery (Figure 3).

**Figure 4:**
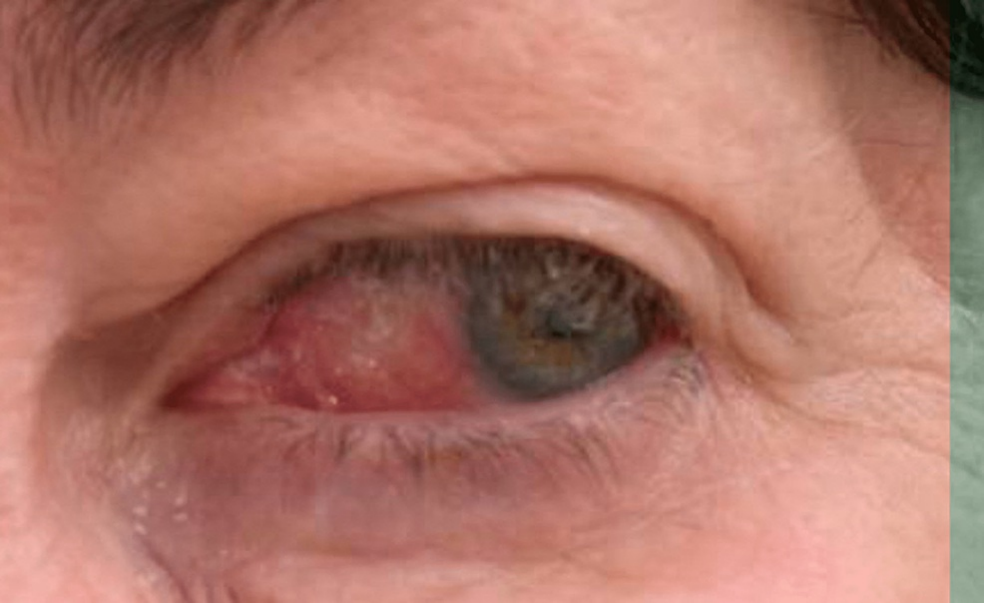
Left eye scleritis after five days of prednisone

The patient received high-dose oral prednisone with a tapered dose and azathioprine, which provided significant improvement within days (Figure 4). She underwent left renal artery angiography and stenting with a good clinical result.

## Discussion

Takayasu arteritis was reported by ophthalmologist Mikito Takayasu in 1908. In the United States, the incidence of TA is approximately 2.6 cases per million people every year. It occurs most commonly in Asian women but has been reported worldwide [4].

The pathogenesis of Takayasu disease is poorly understood. Cellular infiltration localizes in the adventitia and outer parts of the media. The inflammation within the vessel can lead to narrowing and occlusion. Involvement of the subclavian artery is the most common.

Diagnosis can be made using the American College of Rheumatology criteria, which were fulfilled in our patient [4-5]. She was found to have symptom onset at age <40 years, limb claudication, asymmetric blood pressure, decreased pulse, bruit over the subclavian artery, and imaging findings of aorta involvement. The management of TA includes glucocorticoid therapy with a slow taper, adjunctive immunosuppressants, and rarely surgical intervention. Prompt diagnosis of TA and treatment is crucial and can reduce morbidity and mortality. Sudden death may occur due to myocardial infarction, stroke, or aneurysmal rupture.

## Conclusions

Takayasu arteritis is a rare debilitating disease. Early diagnosis is important to improve patient outcomes. In this case, an episode of scleritis led to a diagnosis of TA approximately 25 years after the onset of initial symptoms. When evaluating a patient with scleritis, one should have a high index of suspicion for a systemic autoimmune disease including vasculitis like TA.
